# Customized Information and Communication Technology for Reducing Social Isolation and Loneliness Among Older Adults: Scoping Review

**DOI:** 10.2196/34221

**Published:** 2022-03-07

**Authors:** Gomathi Thangavel, Mevludin Memedi, Karin Hedström

**Affiliations:** 1 Centre for Empirical Research on Information Systems Örebro University School of Business Örebro Sweden

**Keywords:** social isolation, loneliness, review, ICT, older adults, customization, mobile phone

## Abstract

**Background:**

Advancements in science and various technologies have resulted in people having access to better health care, a good quality of life, and better economic situations, enabling humans to live longer than ever before. Research shows that the problems of loneliness and social isolation are common among older adults, affecting psychological and physical health. Information and communication technology (ICT) plays an important role in alleviating social isolation and loneliness.

**Objective:**

The aim of this review is to explore ICT solutions for reducing social isolation or loneliness among older adults, the purpose of ICT solutions, and the evaluation focus of these solutions. This study particularly focuses on customized ICT solutions that either are designed from scratch or are modifications of existing off-the-shelf products that cater to the needs of older adults.

**Methods:**

A scoping literature review was conducted. A search across 7 databases, including ScienceDirect, Association for Computing Machinery, PubMed, IEEE Xplore, PsycINFO, Scopus, and Web of Science, was performed, targeting ICT solutions for reducing and managing social isolation and loneliness among older adults. Articles published in English from 2010 to 2020 were extracted and analyzed.

**Results:**

From the review of 39 articles, we identified 5 different purposes of customized ICT solutions focusing on reducing social isolation and loneliness. These were *social communication*, *social participation*, a *sense of belonging*, *companionship*, and *feelings of being seen*. The mapping of purposes of ICT solutions with problems found among older adults indicates that increasing social communication and social participation can help reduce social isolation problems, whereas fulfilling emotional relationships and feeling valued can reduce feelings of loneliness. In terms of customized ICT solution types, we found the following seven different categories: *social network*, *messaging services*, *video chat*, *virtual spaces or classrooms with messaging capabilities*, *robotics*, *games*, and *content creation and management*. Most of the included studies (30/39, 77%) evaluated the usability and acceptance aspects, and few studies (11/39, 28%) focused on loneliness or social isolation outcomes.

**Conclusions:**

This review highlights the importance of discussing and managing social isolation and loneliness as different but related concepts and emphasizes the need for future research to use suitable outcome measures for evaluating ICT solutions based on the problem. Even though a wide range of customized ICT solutions have been developed, future studies need to explore the recent emerging technologies, such as the Internet of Things and augmented or virtual reality, to tackle social isolation and loneliness among older adults. Furthermore, future studies should consider evaluating social isolation or loneliness while developing customized ICT solutions to provide more robust data on the effectiveness of the solutions.

## Introduction

### Background

Advancements in science and various technologies have resulted in people having access to better health care, a good quality of life, and better economic situations, enabling humans to live longer than ever before. It is estimated that the number of older adults (aged ≥65 years) in the population, as of 2020, is approximately 727 million, and this number is expected to increase to 1.5 billion, which will be approximately 16% of the world’s population by 2050 [1]. Humans are *social beings*; we are biologically and psychologically hardwired to stay connected and be social with other people. If this *socialness* is taken away from us, it can lead to social isolation and loneliness. According to De Jong Gierveld et al [2], loneliness is defined as the subjective feeling of being alone, whereas social isolation is the objective lack of social connections with other people. This review focuses on both social isolation and loneliness.

Studies show that the problems of loneliness and social isolation are much more common among older adults because of various factors such as living alone, the loss of family members or friends, chronic illness, and physical conditions. [3,4]. Furthermore, the recent COVID-19 pandemic has, without doubt, affected people belonging to all age groups. The COVID-19 quarantine restrictions have changed people’s daily lives, resulting in reduced social interaction and social participation [5]. As a result, there has been an increased focus on social isolation and loneliness in all ages, especially in older adults [6]. Both social isolation and loneliness affect people psychologically by increasing stress, anxiety, depression, dementia, Alzheimer disease, cognitive decline, and the risk of suicide [7-9]. In addition, the same studies show that social isolation and loneliness affect people biologically by increasing the risk of many health conditions such as high blood pressure, a weakened immune system, obesity, heart disease, and death. Therefore, with this ongoing pandemic and social distancing norms, there is a need for everyone (not least older adults) to stay connected to prevent, reduce, and manage social isolation and loneliness.

Information and communication technology (ICT) can play an important role in alleviating social isolation and loneliness [10-12]. Social networking services such as Facebook and WhatsApp focus on connecting users with their family or friends and enhancing social relationships. However, the existing commercial off-the-shelf (COTS) products or applications such as Facebook mostly cater to the younger generation and do not consider the needs of older adults [13,14]. As a result, older adults find it difficult to adapt and use these technologies [15]. Therefore, there is a necessity to design customized solutions for older adults that are either designed from scratch or modifications of existing COTS products or applications tailored to the needs of individuals or groups. Although there have been some attempts to design and develop customized ICT solutions that are catered to older adults for managing social isolation or loneliness [16], there is a need to summarize current empirical research on these customized ICT solutions to understand what the existing ICT solutions provide and what purpose they have. Currently, there are some literature reviews summarizing ICT solutions (see the *Related Research* section below) that address social isolation or loneliness for older adults. However, to the best of our knowledge, there is no literature review that summarizes only customized ICT solutions that are designed for older adults for reducing social isolation or loneliness.

In addition, in general, there are different mechanisms and purposes in developing solutions that target social isolation and loneliness [17,18]. For example, messaging applications focus on improving social communication among older adults to reduce their social isolation. In addition, recent technology has developed a long way and introduced social robots for older adults that provide companionship to combat loneliness [19]. Therefore, finding out the purpose of each ICT solution will help in choosing the appropriate solution for managing social isolation or loneliness. Hence, to address this gap, this review summarizes the customized ICT solutions that are designed for older adults for reducing social isolation and loneliness. In addition, this review investigates the purpose of each ICT solution and the evaluation focus of these solutions.

The following research questions have been identified and addressed in this study: (1) What were the purposes of the customized ICT solutions for reducing social isolation or loneliness? (2) What are the customized ICT solutions proposed for reducing social isolation or loneliness among older adults? (3) What aspects of customized ICT solutions have been evaluated?

This study updates the existing literature with the latest evidence on ICT solutions, focusing on social isolation or loneliness among older adults. This review also intends to provide practitioners and researchers in this field with a better insight into how to manage social isolation or loneliness among older adults by distinguishing different types of ICT solutions, the purposes of these solutions, and what has been evaluated.

### Related Research

In this section, we will present previous literature reviews that exclusively investigated ICT interventions targeting social isolation or loneliness among older adults to position our literature review and knowledge contribution. Below, Table 1 provides a summary of the current literature reviews, describing the years the literature review covers, the problems investigated, whether the literature review included customized solutions, the purpose of the ICT intervention, and the types of ICT interventions identified in their studies.

As shown in Table 1, 6 reviews provided empirical evidence of ICT solutions for reducing loneliness or social isolation among older adults. However, they mostly covered general ICT use, computer training, and existing social network impact and included less customized solutions or did not include them at all [11,12,20-23]. The review by Baker et al [10] included studies with existing COTS applications as well as small-scale studies that developed prototypes or applications. They addressed social isolation and focused on solutions that increased social participation. In the same review, they found that social networking services (5 COTS and 8 customized) and *touch screen*–based interventions (1 COTS and 8 customized) were primarily used to combat social isolation. None of the reviews in Table 1 covered only customized solutions that were designed specifically for older adults. In addition, the purposes of the ICT interventions were not examined explicitly in those reviews.

**Table 1:** Overview of existing literature reviews of information and communication technology (ICT) interventions.

Source	Years included in the review	Problem investigated	Included customized solutions	Purpose of ICT intervention covered (Research question 1)	Types of ICT interventions (part of research question 2)
Baker et al [10]	2000 to August 2016	Social participation and reducing social isolation	Yes (23 out of 36 included studies)	Not covered	Touch screen technology Social networking services Adaptation of existing technology platforms Use of games ICT training
Chen and Schulz [11]	2002 to 2015	Social isolation (but included both loneliness and social isolation)	Yes (3 out of 25 included studies)	Not covered	General ICT use Social networking services Telephone befriending Video games Virtual pet
Ibarra et al [12]	Until January 2020	Social isolation and loneliness	Yes (10 out of 25 included studies)	Not covered	General internet use for interaction (eg, discussions in forums) and email Video chat Social networks Virtual spaces or classrooms with messaging capabilities Messaging services Virtual companions Phone calls
Khosravi et al [20]	2000 to 2015	Social isolation and loneliness	Yes (6 out of 34 included studies)	Not covered	General ICT use Video game Robotics Personal reminder information and social management system Asynchronous peer support chat room Social networking sites Telecare 3D virtual environment
Noone et al [21]	2004 to April 7, 2020	Social isolation and loneliness	Not included	Not covered	Only 1—videoconferencing
Choi et al [22]	2001 to July 2012	Loneliness	Not included	Not covered	Not categorized but included only computer training and general ICT use studies
Casanova et al [23]	2002 to 2019	Loneliness	Not included	Not covered	Not categorized but included only general ICT use, computer training, and social network studies

## Methods

### Overview

This paper undertakes a scoping review of the literature to summarize the customized ICT solutions for reducing social isolation or loneliness among older adults. We conducted a scoping literature review in which the mnemonic population, concept, and context guided the focus [24,25]. The population in question was older adults. The concept related to customized ICT solutions proposed for reducing social isolation or loneliness among older adults. The context was the setting; that is, where these types of ICT solutions are being used (eg, in a private home or in a nursing home). The review process started with planning the review protocol and continued with a search process, practical screening of articles, extraction, and analysis of data.

### Search Strategy

Electronic searches for this study were conducted in December 2020 using the following seven databases: ScienceDirect, Association for Computing Machinery, PubMed, IEEE Xplore, PsycINFO, Scopus, and Web of Science. In this study, the Association for Computing Machinery and IEEE Xplore were chosen to cover computing and information technology articles, whereas PubMed and PsycINFO were chosen to include medical and psychology-related articles. ScienceDirect, Web of Science, and Scopus were selected to include studies in multidisciplinary areas of interest such as the social sciences. On the basis of the research questions, keywords were divided into three categories: older adults (“Older” OR “Senior” OR “Elder”), technology intervention (“Information and Communication technology” OR “ICT” OR “Internet” OR “Mobile” OR “Sensor” OR “Social media” OR “Information technology” OR “HCI” OR “Human Computer Interaction” OR “Robot” OR “Computer”), and problem (“Loneliness” OR “Social isolation”). The search was limited to publication years (2010 to 2020); *title, abstract, and keywords*; and articles in English. The initial search process was carried out by the first author (GT). The inclusion and exclusion criteria and data extraction format were drafted by the first author and then reviewed and finalized in coordination with the coauthors. Following that, screening and data extraction were performed by the first author and, in case of any uncertainty, the coauthors were consulted. Further conflicts were resolved through discussion until a consensus among the authors was reached.

### Inclusion and Exclusion Criteria

We defined the following inclusion and exclusion criteria (Textbox 1) to retrieve the eligible studies for this review. An article was retained if it met all the inclusion criteria and was rejected if it met any of the exclusion criteria.

**Inclusion and exclusion criteria.**

**Inclusion criteria**

- Full paper written in English
- Empirical studies that developed or presented a customized information and communication technology (ICT) solution with a primary focus on loneliness or social isolation
- Focus on older adults (as defined by the articles)

**Exclusion criteria**

- Nonresearch articles (such as magazines, guest editorial letters, forewords, keynotes, book reviews, posters, and workshop invitations)
- Empirical articles that did not develop or present a customized ICT solution for managing loneliness or social isolation
- Conceptual studies, theoretical studies, or review articles

### Selection of Articles

**Figure 1:**
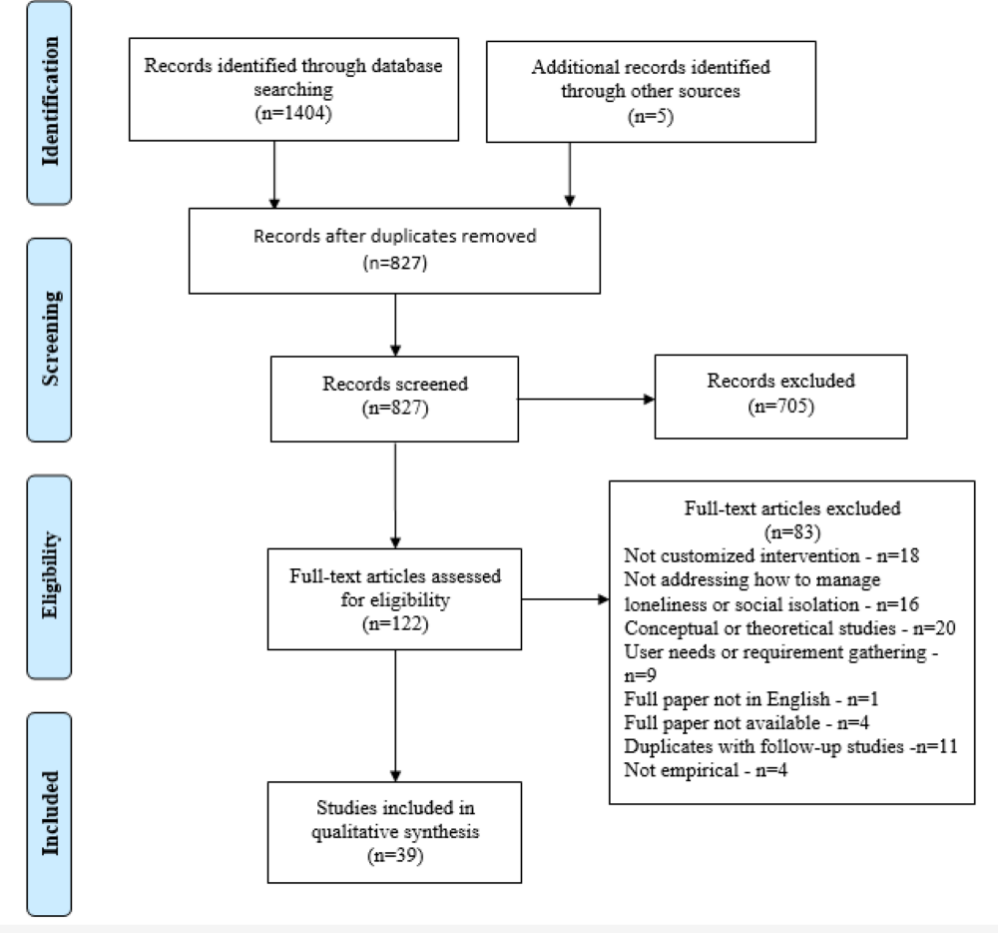
Overview of the selection process based on the PRISMA (Preferred Reporting Items for Systematic Reviews and Meta-Analyses) statement [26].

Using the above-mentioned search strategy, 1409 articles were retrieved—584 (41.45%) from Web of Science, 338 (23.99%) from Scopus, 188 (13.34%) from PubMed, 97 (6.88%) from PsycINFO, 83 (5.89%) from the Association for Computing Machinery, 72 (5.11%) from IEEE, and 42 (2.98%) from ScienceDirect, and 5 (0.35%) additional articles were retrieved through a manual search by screening reference lists. Subsequently, 41.31% (582/1409) duplicate articles were removed. The remaining 827 articles were screened by reading their abstracts, which resulted in 122 (14.8%) papers that fit the focus and scope of this study. Full texts of the 122 studies were then screened using the inclusion and exclusion criteria (Textbox 1), and 72 (59%) papers were excluded. Furthermore, if an author published multiple follow-up articles with the same ICT solution, the most recent article or the one with most of the details was selected. In this way, another 9% (11/122) of articles were excluded, which led to 39 articles being retained. The entire selection process and the reasons for exclusion are outlined and reported in Figure 1 based on the PRISMA (Preferred Reporting Items for Systematic Reviews and Meta-Analyses) statement [26].

### Data Analysis and Synthesis

From the shortlisted articles, the first author extracted the following data based on the research questions. To answer the first research question (ie, the intended purpose of the proposed ICT solution), we extracted the problem area and aim of the proposed solution. If the aim was not mentioned explicitly or was not clear, we analyzed the solution and noted what the solution was set to accomplish. For the second research question (ie, customized ICT solutions), we obtained the types of ICT solutions based on the use or functionality, the devices used, and their features. In addition, adapted from another review study [12], if the solution facilitated social interaction, we noted a different context of interactions and the contacts made by the participants. We used the existing categories (ie, social network, video chat, messaging services, video games, robotics, virtual spaces, or classrooms with messaging capabilities) identified in previous reviews [12,20] as a starting point to categorize the ICT solutions and added if any new type of ICT solution was found. For example, we added *content creation and management system*. For the third research question, we noted how the study was evaluated, its sample size, the age group of the included participants, the evaluation environment, the dependent variables, the scales used for the dependent variables, and the outcome of those measurements. Owing to the heterogeneity of the included studies, narrative synthesis was performed.

## Results

### Main Characteristics of the Included Studies

Most of the 39 included studies were conducted in Europe (20/39, 51%), followed by North America (9/39, 23%) and Asia (4/39, 10%). Nearly 16% (6/39) of the included studies lacked information about their country of study. The age group of the study population varied among the studies. Most of the studies (16/39, 41%) included older adults with a starting age of 65 years, followed by ≥60 years (8/39, 21%), ≥55 years (4/39, 10%), ≥70 years (3/39, 8%), ≥75 years (2/39, 5%), and ≥50 years (2/39, 5%), and the remaining studies did not mention the starting age of their study population. The customized solutions designed by the included studies were mostly tested in regular living environments (28/39, 72%), such as older adults’ homes, care centers, retirement homes, and nursing homes. Of the 39 studies, 6 (15%) tested their solutions in their laboratory environments, 1 (3%) tested it in a hospital setting, another one (3%) evaluated it in an exhibition setting, and 3 (8%) did not provide information about the study environment.

From the review of 39 studies, most (20/39, 51%) addressed the problem of social isolation (Multimedia Appendix 1 [12,16,27-63]), whereas 9 (23%) focused on loneliness, and 10 (26%) focused on both social isolation and loneliness. Studies that mentioned both problems considered social isolation and loneliness as similar concepts and referred to them interchangeably. For instance, Sidner et al [27] referred to social isolation in their aim and used *loneliness* in the hypothesis, whereas Goumopoulos, Papa, and Stavrianos [28] used both *social isolation* and *loneliness* in their aim and hypothesis but measured only loneliness. Some studies defined the concept of social isolation as a lack of social relationships, a lack of social support, and reduced participation in social activities [29-31]. Of the 39 studies, 2 (5%) pointed out that, because of the high use of digital communication among younger generations, older adults who were reluctant to use or uncomfortable with existing technologies were not able to have social interactions and felt left out, which contributed to their social isolation [32,33]. The studies addressing loneliness defined it as “feeling invisible,” solitude, and living alone [34-36]. Overall, 33% (13/39) of the studies did not have a clear definition of these 2 concepts.

### Purposes of Customized ICT Solutions

**Figure 2:**
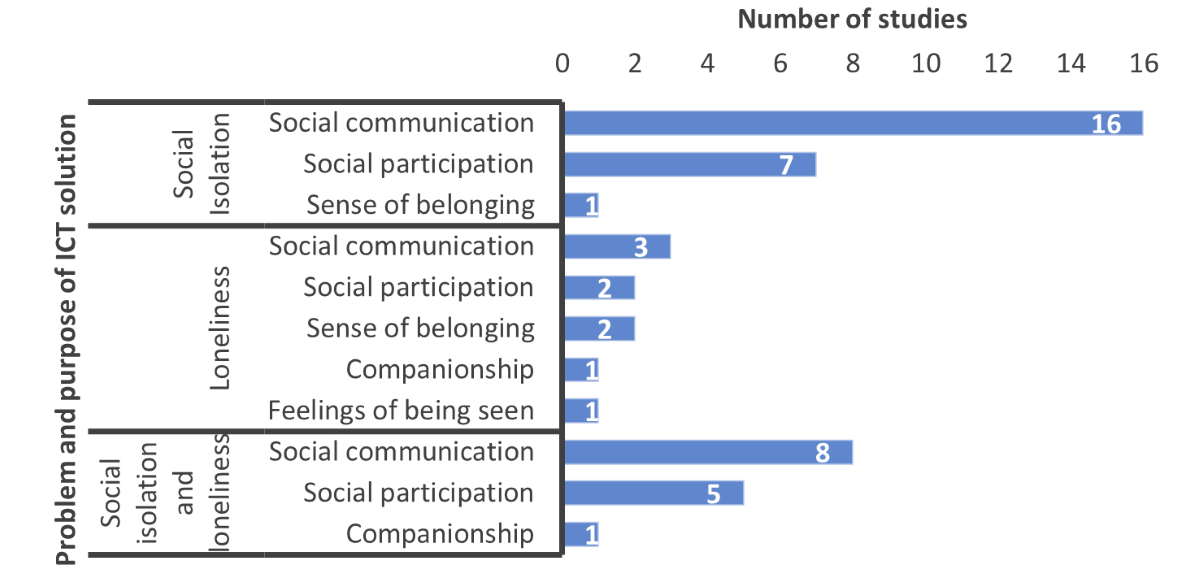
Problems addressed and purposes of the ICT solutions. ICT: information and communication technology.

With respect to the purposes of the proposed solutions, 5 different purposes were identified from the included studies. Most of the studies (31/39, 79%) focused on 1 purpose and designed the solution based on this, whereas 21% (8/39) focused on 2 purposes and provided solutions. The problems addressed in the included studies and the purposes of the proposed solutions are shown in Figure 2. Most of the studies (27/39, 69%; Multimedia Appendix 1) addressed the problem of social isolation or loneliness with the purpose of increasing *social communication* (ie, increasing the older adult’s social interaction with their social contacts, mainly family and friends, through web-based chat, videoconferencing, group chat, and email [28,33,37]). Few studies provided the facility to interact with other contacts such as physicians or nurses [38] or with a virtual coach or helper [36,39,64]. One particular study (1/39, 3%) created a virtual coach application that helped and encouraged older adults to interact and make friends with strangers [36].

The second most common purpose was *social participation* (14/39, 36%). Here, the studies focused on engaging older adults with web-based activities such as gaming [40-42] or participating in web-based exercise [43,64] or art making [44]. Few studies focused on discussing shared interests or hobbies either in a virtual class environment [45] or by forming a small social network group [29,30]. Other studies encouraged social participation by stimulating older adults to visit local social activities [28,46,47] or family events [48] or go shopping [49].

Increasing the *sense of belonging* was emphasized by a few studies to make older adults feel part of broader society, enhance their self-esteem, and feel valued [35,50,51]. For this, those studies created a platform to share user-generated content such as life experiences [35], memories [51], and cooking recipes [50] to pass on to next generations.

Overall, 5% (2/39) of the studies described the purpose as *companionship* to fulfill older adults’ emotional relationships by providing virtual support with the help of robots and companionable agents [27] or through a virtual pet application [52].

In total, 3% (1/39) of the studies focused on increasing the feelings of *being seen* to create a sense of care and fulfillment among older adults [34].

### Types of ICT Solutions

#### Overview

We found 7 different types of ICT solutions developed based on the purposes discussed in the previous section to answer the second research question. These were *social networks*, *messaging services*, *video chat*, *virtual spaces or classrooms with messaging capabilities*, *robotics*, *games*, and *content creation and management*. Few studies implemented combinations of these technologies in their solutions. Social networks were mainly proposed by many studies (14/39, 36%), followed by video chat (8/39, 21%), messaging services (8/39, 21%), robotics (6/39, 15%), virtual spaces or classrooms with messaging capabilities (7/39, 18%), games (4/39, 10%), and content creation and management (2/39, 5%). In these different types of solutions, a *tablet* device was the most used for implementation (16/39, 41%). Other devices used in the studies were televisions (8/39, 21%), desktops (6/39, 15%), customized robots or new robotic objects (6/39, 15%), smartphones (4/39, 10%), sensors (2/39, 5%), and a patented communication device named *ippi* [53] (1/39, 3%). Achilleos et al [45] created a customized device using existing devices such as the Mac mini, camera, and microphone, whereas Garattini et al [54] created a novel “building bridges device” using a touch screen computer connected to a custom-made stand along with a phone handset. Similarly, an Android phone was turned into a Raspberry-like board to create a memory music box [55]. Approximately 5% (2/39) of the studies used tangible interfaces such as a flower vase with a microphone [31] or a glass window with a television screen, camera, and microphone [56].

In terms of customization, a few created customized devices as mentioned above. Some created new applications with a customized interface [30,32]. In this regard, most of the studies developed the interface with simple and easy-to-use menus and layouts [16,32,57]. Some studies considered age-appropriate usability and accessibility guidelines based on previous literature or as per older adults’ input (eg, voice typing; offering large, nontextual touch icons; audio messages; voice commands; and easy navigations [32,33,35,58,59]). In addition, some studies considered the television as an alternative to the desktop or tablet. For interactions, the user could either use a remote control or speech and gestures [60]. For individual privacy concerns, 3% (1/39) of the studies added avatar features for the older adult group exercise application, which can help reduce physical barriers and get users more engaged in the activities [64]. In addition, 5% (2/39) modified the existing social network interface and made privacy options simple and easy to understand [16,60].

#### Social Networks

Most of the studies (14/39, 36%) proposed social networks as a solution to overcome social isolation or loneliness problems by connecting older adults with their social contacts. There were 2 different types of social networks designed. Some studies created a *user-friendly tailored interface to access existing social networks* such as Facebook [16,28,32,60-62], Twitter [62], Instagram [33], and YouTube [35]. All the studies presented social media content from friends and family in a way suitable for older adults. In the above studies, except for Romanyk et al [62] and Tapia et al [33], the customized social networks allowed the user to create posts and share them with their network. The interfaces mostly used either the *tablet* or *television* to display the social media content to the user.

Another set of studies *created a new social network* to build a virtual community and participate in social activities [29,30,46,47,49,57]. Buhr et al [30] built a social network specifically for individuals with aphasia to share their personal stories and information about *living with aphasia*. In the same way, FridgeNet, a network created by Lee et al [49], focuses on the topic of a healthy diet, and users can share diet information with their peers. This also encourages users to meet face-to-face by sending a shopping invitation and purchasing food together. Approximately 5% (2/39) of the studies motivated older adults to meet friends or neighbors face-to-face who reside in the same community for participating in social activities [46,47].

#### Video Chat

The second most used technology by the studies (8/39, 21%) in their solutions was video chat to boost social interaction, mainly with family or friends. Video chat was mostly proposed as an additional social feature along with other solutions such as games and messaging services. Of the 39 studies, 3 (8%) developed video chat as the primary solution. Pereira et al [59] designed a smart remote-control application that can control the television as well as make video calls using voice commands. Approximately 5% (2/39) of the studies proposed a simple interface for video communication that can be used by older adults without much assistance. Angelini et al [56] used glass windows to establish a permanent video connection with a distant relative by opening the blind of a window screen. In a similar way, Kleinberger et al [55] created a memory music box. When older adults open the box, a photo slideshow is played and a notification is sent to their grandchild via email automatically. They can then make a video call, if available.

#### Messaging Services

Messaging services were used as a solution to enhance the social interactions of older adults, mainly with their family or friends. The different messaging services used in those studies were SMS text messages, voice messages, video messages, email, and photos. The interactions enabled through these messaging services were one-to-one communication in most of the studies except Garattini et al [54], who introduced group chat options. The study by Zaine et al [63] introduced a web-based application in which a human facilitator places a time-based request, collects the media message, and distributes it to a target person with a text commentary to deepen existing relationships.

#### Virtual Spaces or Classrooms With Messaging Capabilities

Virtual spaces or classrooms are web-based spaces where older adults form a group to discuss their interests and that provide opportunities to participate in voluntary activities such as teaching other older adults [40,45]. Approximately 5% (2/39) of the studies implemented web-based live exercise classes in which older adults could interact with instructors and other members virtually [43,64]. A “virtual coaching” application was proposed by 5% (2/39) of the studies, providing friendship enrichment lessons to encourage older adults to make new friends by giving them tasks such as “go for a walk with someone” [36,39]. Another study (1/39, 3%) created a *virtual companion* in the form of a web-based pet application mediated by a human helper to provide companionship by monitoring the older adults visually, having deep conversations, and contacting caregivers in case of an emergency [52].

#### Robotics

Robotics technology provides emotional support by using nonverbal gestures, which increase the feelings of *being seen* to reduce loneliness [34]. Robots talk with older adults about different topics and connect them with their family or friends to provide companionship and increase social interaction [27,38,48]. In addition, robots help older adults engage in activities to keep them occupied, such as listening to music and playing games [27,41,48], or involve them in participatory arts such as reciting Shakespeare sonnets [44].

#### Games

Of the 39 studies, 2 (5%) primarily proposed games to entertain older adults and reconnect with other persons who participate in the games. Doppler et al [42] created an application for card games with a videoconferencing function to facilitate older adults’ interaction while playing. Similarly, Correia et al [41] developed a robot and a touch interface to play card games with older adults as a team player and also as an opponent. Other studies considered games as secondary solutions in their applications.

#### Content Creation and Management System

Approximately 5% (2/39) of the studies developed simple and easy-to-use web applications, which had built-in templates that helped older adults create content. The NoBits application allowed older adults to capture and upload their memories, local history personified by photos, newspapers, and postcards. [51]. Similarly, the application developed by Tullius and Dogan [50] encouraged older adults to create and share food recipes to help people who were in need.

### Evaluation Focus of Customized ICT Solutions

#### Overview

In terms of evaluations, all the studies except 1 [41] were evaluated with older adults. Correia et al [41] tested their game with younger participants. Most of the included studies (21/39, 54%; Multimedia Appendix 2 [12,16,27-63]) measured the *usability* of the developed solution. In addition, of the 39 studies, 11 (28%) reported the users’ acceptance of their solutions; 11 (28%) examined loneliness; and 6 (15%) evaluated social isolation in the form of social support, social engagement, and social connectedness. Few studies also analyzed and reported use, general feedback about the solution, self-perceived health, quality of life, depression, emotional well-being, and self-esteem.

#### Usability and User Experience

Approximately 14% (3/21) of the studies carried out *heuristic evaluation* with experts initially to determine the usability problems [29,33,45]. The suggestions were implemented and evaluated further with the older adults. Of the 21 studies which measured usability, 6 (29%) measured with the System Usability Scale, 2 (10%) used the User Experience Questionnaire, and 1 (5%) used the Computer System Usability Questionnaire [51]. Almost all the studies that reported using these scales showed positive results and high ratings [29,45,60,64]. Only 5% (1/21) of the studies reported a below-average score of 65.3 on the System Usability Scale [46]. Older adults perceived the ICT interventions as easy to use and found them useful [29,32,40,53]. For some studies, it was initially difficult for older adults to use the services immediately, but training and support or use over time helped them gain confidence, which later improved their usability at the end of the study period [58,64]. The participants also rated the overall *user experience* [46,60,63] as high except for one of the studies [36], where participants were missing some fun in using the system.

#### Users’ Acceptance of the Solutions

Of the 39 studies, 11 (28%) reported the user acceptance and attitudes toward the developed solutions. The results were mostly positive, and the participants were willing to use the system in the future [28,47,53]. Older adults perceived the system as useful and saw potential to improve their social connectedness [55]. Technology satisfaction significantly increased, and there was a significant difference between control and intervention over time [64]. In addition, the participants gave a positive opinion about using new technology solutions [50].

#### Loneliness and Social Isolation

Loneliness was measured using the University of California, Los Angeles Loneliness Scale in 45% (5/11) of the studies that measured loneliness, and 18% (2/11) used the revised version of the same scale. Jansen et al [46] used the De Jong Gierveld and Kamphuis 11-item loneliness scale, whereas Brandenburgh et al [39] used the short version of the same scale that comprises 6 items. Morganti et al [51] used the Italian Loneliness Scale, which has 18 items that are grouped into three subscales: emotional loneliness, social loneliness, and general loneliness. In total, 3 (27%) studies conducted randomized controlled trials in which there was a significant decrease in loneliness among older adults who used the intervention compared with the control group [40]. There was a reduction in loneliness in other studies [51,64], but there was no significant difference when compared with the control group. This was because there was constant contact with the coach over the phone in the exercise program [64], whereas the older adults in the control groups were doing a reminiscing activity with a children group in Morganti et al [51]. Other 8(73%) pre-post, quasi-experimental, and mixed methods studies had a varied response. Of the 8 studies, 4 (50%) resulted in a reduction in loneliness among older adults [39,43,44,52], whereas Goumopoulos et al [28] showed a moderate improvement in reducing loneliness, and 3 other studies (38%) [27,37,46] reported that there was no significant change in loneliness.

Social isolation was examined using different measurement scales such as the Friendship Scale, Lubben Social Network Scale, Duke Social Support Index, and Norbeck Social Support Questionnaire. Social interactions and social engagement increased after a period of use [43,54,63]. There was a significant decline in social isolation and an increase in social support [40]. In contrast, in 5% (2/39) of the studies, there were no significant changes in the participants’ social relationships or interactions [27,37].

#### Health-Related Outcomes

Health and health-related quality of life were analyzed in 10% (4/39) of the studies, of which 50% (2/4) showed an improvement in health [40,46], and 50% (2/4) reported no significant changes in health [27,58]. Approximately 5% (2/39) of the studies examined the status of depression and, in both, depression was reduced [43,44]. Emotional well-being improved [43]. The outcome of self-esteem only increased in the control group and did not increase in the intervention group [51].

## Discussion

### Principal Findings

In relation to previous reviews (Table 1), which mostly examined general ICT use or existing ICT interventions, this study reviewed only customized ICT solutions that were designed and developed for older adults to manage social isolation or loneliness. The analysis of the reviewed studies highlights a growing interest in applying customized ICT solutions for reducing social isolation or loneliness among older adults. The results underline the need to increase the aspects that contribute to reduced social isolation or loneliness among older adults using ICT solutions. Such aspects include *social communication*, *social participation*, a *sense of belonging*, *companionship*, and *feelings of being seen*. The studies mostly focused on increasing social communication followed by social participation to build solutions for managing social isolation. Here, *social communication* helps older adults increase their social connection with their family or friends, and *social participation* facilitates engagement in social activities with others. The studies that focused on loneliness alone (9/39, 23%) included aspects such as sense of belonging, companionship, and feelings of being seen apart from increasing social communication or participation [34,52]. Here, even though the purpose of *social communication* is the same, an emotional parasocial relationship was provided by a virtual coach [36]. Similarly, the other 3 purposes—a sense of belonging, companionship, and feelings of being seen—concentrate on fulfilling emotional relationships.

This mapping of problems (social isolation and loneliness) with the purposes of ICT solutions shows the important differences between the solutions for managing social isolation and loneliness among older adults, which was not highlighted in previous reviews. The results indicate that increasing social communication and social participation can help reduce the social isolation problem, whereas fulfilling emotional relationships and feeling valued can offer support in tackling loneliness. These results also highlight the importance of discussing and managing social isolation and loneliness as different but interrelated concepts and also emphasize the need for future research to use a suitable outcome measure for evaluating the ICTs based on the problem they address.

Seven different types of customized ICT solutions were identified: *social networks*, *messaging services*, *video chat*, *virtual spaces or classrooms with messaging capabilities*, *robotics*, *games*, and *content creation and management*. In contrast to previous reviews [11,12,20], which mostly reported general ICT use such as the use of computers and the internet, it was found that social networks were common in the reviewed studies, which is in line with the review by Baker et al [10]. Previous reviews [11,12,20] have included mostly off-the-shelf solutions and less customized solutions, which can explain this result. In terms of the devices used or suggested in the proposed solutions, *tablet* was the most preferred, followed by *television*. The choice of *tablet* for older adults is because of the portability and usability options it provides, whereas the television medium is widely adopted because users feel more comfortable and familiar as it has been present in almost all houses for several years [65-67]. Even though technology has widely advanced in terms of the Internet of Things and virtual or augmented reality, there were no studies in our review proposing their use. This highlights the need for future research to explore emerging technologies to reduce social isolation and loneliness.

Going further, most of the included studies evaluated the usability and acceptance aspects of the ICT solutions, and fewer studies focused on loneliness or social isolation outcomes. Training and support were shown to be important factors in achieving a greater usability score, which is in line with the results of the review by Ibarra et al [12]. In addition, older adults who perceived the application as useful were more willing to use the system in the future. Studies that evaluated loneliness or social isolation outcomes mostly reported a positive response in reducing loneliness or social isolation, but only 8% (3/39) of the studies were assessed using a randomized controlled trial design. In addition, the included studies in this review examined different outcomes by using several measurement scales or used qualitative methods and reported the outcomes in various ways. Owing to the heterogeneity of the included studies, we were not able to assess the effectiveness of each customized ICT solution. Future studies should consider evaluating social isolation or loneliness along with other outcomes while testing their customized ICT solutions.

### Strengths and Weaknesses of This Review

This is the first review that had a clear focus on customized ICT solutions targeting social isolation or loneliness. This review highlighted the differences between the solutions for managing social isolation and loneliness. Although a significant effort was made to ensure the rigor of the search strategy, potentially relevant studies may not have been identified if the authors did not use the search keywords that were included in this review. In addition, limiting the search to include articles published in the English language may also have omitted additional relevant studies in other languages.

### Conclusions

This scoping review summarized the customized ICT solutions designed for older adults for managing social isolation or loneliness. In addition, this review investigated the purpose of each ICT solution and the evaluation focus of these solutions. The mapping of social isolation and loneliness problems and the purpose of ICT solutions shows the important differences between the solutions targeting social isolation and loneliness. In terms of ICT solutions, we found 7 different categories, of which social networks were the most proposed. Furthermore, this review highlights the importance of discussing and managing social isolation and loneliness as different but related concepts and emphasizes the need for future research to use suitable outcome measures for evaluating the interventions based on the problem. Even though a wide range of customized ICT solutions have been developed, future studies need to explore recent emerging technologies such as the Internet of Things and augmented or virtual reality to tackle social isolation and loneliness among older adults. Finally, future studies should consider evaluating social isolation or loneliness while testing customized ICT solutions.

## Appendix

Multimedia Appendix 1 Details of the included studies.

Multimedia Appendix 2 Details of the interventions’ dependent variables and their outcomes.

## Abbreviations

COTS: commercial off-the-shelf
ICT: information and communication technology
PRISMA: Preferred Reporting Items for Systematic Reviews and Meta-Analyses
