# Supplementary material for: Customized Information and Communication Technology for Reducing Social Isolation and Loneliness Among Older Adults: Scoping Review
Source: JMIR Ment Health. 2022 Mar 7;9(3):e34221. doi: 10.2196/34221 (PMC8938833; doi:10.2196/34221)
Supplement: Multimedia Appendix 1 [file mental_v9i3e34221_app1.pdf]

Multimedia Appendix 1

Details of included studies (n=39).

Source	Problem discussed in thestudy	Purpose of intended solution	Type of technology	Devices	country ofstudy
Achilleos et al. (2013)	Social isolation	increase social communication and social participation	virtual spaces or classrooms with messaging capabilities, video chat	created a customised device with 2 Mac mini,2 cameras and microphone	did not mention
Alaoui & Lewkowicz (2013)	Social isolation	increase social communication and social participation	Social network	TV, Tablet	France
Angelini et al. (2016)	Social isolation	increase social communication	Video chat	Glass window with a TV screen camera and microphone	Switzerland
Báez et al. (2016)	Social isolation	increase social participation and social communication	virtual spaces or classrooms with messaging capabilities, video chat	Tablet and necklace sensor	Italy
Boyd et al. (2015)	Social isolation	increase social communication	Social network	Tablet	UK
Brandenburgh et al. (2014)	Loneliness	increase social communication – para social relationships	Virtual coaching	Tablet, Desktop, Sensor	Netherland
Buhr et al. (2017)	Social isolation	increase social participation	Social network	Tablet (iPad)	USA
Caroux et al. (2017)	Social isolation	increase social communication	Messaging services (Email)	Tablet	did not mention
Casey et al. (2020)	Social isolation and Loneliness	increase social participation and social communication	Robotics	Robot	UK, Italy and Ireland
Coelho et al. (2017)	Social isolation	increase social communication	Social network	TV, Kinect camera, Desktop, and remote control	Portugal
Correia et al.(2016)	Social isolation	increase social participation	Games	Robot	Portugal

Czaja et al. (2018)	Social isolation and Loneliness	increase social participation and social communication	virtual spaces or classrooms with messaging capabilities, Games	Desktop	USA
Doppler et al.(2018)	Social isolation	increase social participation and communication	Games, Video chat	TV, Tablet	Austria
Fields et al. (2019)	Loneliness	increase social participation	Robotics	Robot	USA
Gao et al. (2015)	Loneliness	increase social participation	Social network	Touch screen phone	China
Garattini et al.(2012)	Social isolation and Loneliness	increase social communication	Messaging service (broadcast, text, group chat)	touch screen computer in a custom-made stand connected with a phone handset with functioning cradle and speakers	Ireland
Gomes et al. (2014)	Social isolation	increase social communication	Social network	Tablet	did not mention
Goumopoul oset al. (2017)	Social isolation and Loneliness	increase social communication and participation	Social network	Desktop, Tablet	Greece
Isaacson et al.(2019)	Social isolation and Loneliness	increase social participation and social communication	virtual spaces or classrooms with messaging capabilities, video chat	TV, remote and webcam	USA
Jansen-Kosterink et al.(2020)	Social isolation and Loneliness	increase social participation	Social network	can be accessible via smartphone, tablet, and desktop computer	Netherland
Kleinberger etal. (2019)	Social isolation	increase social communication	Video chat	Android device processor turned into a Raspberry- like board	USA
Koceski & Koceska (2016)	Social isolation and Loneliness	increase social communication	Robotics	Robot	Macedonia

Lee et al. (2015)	Social isolation	increase social participation	Social network	Tablet	did not mention
Machesney, Wexler, Chen, & Coppola, (2014)	Loneliness	companionship - emotional interaction	Virtual companion	Tablet	USA
Marcelino et al. (2016)	Loneliness	increase sense of belonging	Social network	Desktop computer	Portugal
Morganti et al. (2016)	Loneliness	increase sense of belonging	Content creation and Management system	Desktop	Italy
Muñoz et al. (2015)	Social isolation	increase social communication	Social network, video chat	Tablet	did not mention
Muuraiskangaset al. (2012)	Loneliness	increase social communication - parasocial relationships	Virtual coaching	Tablet	Netherland
Neves, Franz, Judges, Beermann, & Baecker (2019)	Social isolation and Loneliness	increase social communication	Messaging service (Video, Audio, Photo)	Tablet (iPad)	Canada
Pereira et al. (2015)	Social isolation	increase social communication	Video chat	Smart phone	Portugal
Restyandito etal. (2020)	Loneliness	increase social communication	Social network	Smart phone	Indonesia
Romanyk et al.(2015)	Social isolation and Loneliness	increase social communication	Social network	TV	Canada
Scandurra & Sjölinder (2013)	Social isolation	increase social communication	Messaging service (Video, Audio, Photos, and text), Games	communication device ippi, TV	Sweden

Sidner et al. (2018)	Social isolation and Loneliness	companionship	Robotics	Robot, Touch screen computer	USA
Tapia et al. (2016)	Social isolation	increase social communication	Social network, email	smart phone and smart TV	did not mention
Tullius & Dogan(2020)	Social isolation	increase sense of belonging	Content creation and Management system	platform independent though intended for tablet	Germany
Zaine et al. (2019)	Social isolation	increase social communication	Messaging services (Video, Audio, Photos, and text)	Smart phone	UK andBrazil
Zhao et al. (2016)	Social isolation	increase social communication	Messaging service (voice message)	Tangible interface - Flower vase with microphone, small single-board computers	Hongkong
Zuckerman etal. (2020)	Loneliness	increase the feelings of "being seen"	Robotics (Non - Humanoid Robot)	Non-Humanoid Robot	Israel
