# Supplementary material for: Customized Information and Communication Technology for Reducing Social Isolation and Loneliness Among Older Adults: Scoping Review
Source: JMIR Ment Health. 2022 Mar 7;9(3):e34221. doi: 10.2196/34221 (PMC8938833; doi:10.2196/34221)
Supplement: Multimedia Appendix 2 [file mental_v9i3e34221_app2.pdf]

Multimedia Appendix 2

Details of Interventions dependent variables and its outcome(n=39).

Source	Study design or Method	Sample size	Age	Environment	Dependent variables (measurement scales)	Outcome
Achilleos et al. (2013)	Heuristic Evaluation - Experts	10 (experts)	NA	Lab	Usability (SUS)	SUS score – 63 (Below average), implemented the changes and evaluated with older adults.
	Mixed methods – Older adults	30	did not mention	Lab	Usability (SUS)<br>User experience and attitude	SUS score – 78 (Above average)<br>most of them especially appreciated communicating via the Meet format
Alaoui & Lewkowicz (2015)	Heuristic Evaluation - Experts	did not mention	NA	Lab	Usability	Experts suggested interface improvements which are then implemented and evaluated further with older adults
	Mixed methods	5	65+	Home	Usability (SUS)	Older adults satisfied with the services<br>SUS score 75 out of 100 (Above average)
Angelini et al. (2016)	Mixed methods (Observation and Interview)	8	65+ (Mean 86.5)	Nursing home	User acceptance	The older adults stated that the system could help them to stay in touch with their family
Báez et al. (2016)<br>Báez et al. (2017)	RCT (Pilot)	37<br>(Basic app for control group– 17, Advanced app for social group- 20)	65+ (Mean 71.2)	Home	Usability (SUS)<br>Technology acceptance<br>Usefulness<br>Loneliness (R-UCLA scale)	-Usability increased at the end of study<br>-technology satisfaction significantly increased from pre- to post- for the advanced but not for the basic group<br>-participants perceived the application as more useful after the training program, and that perceived usefulness in the social group may have improved more with respect to the control group.<br>-No significant difference in loneliness
Boyd et al. (2015)	Comparative study (Facebook and Easisocial) with same participants	9	65+ (Mean age 76.5)	Home	Usability -usefulness, ease of use, ease of learning and satisfaction	-No significant difference in satisfaction (p = 0.5404) and usefulness (p = 0.1735) but a significant difference in ease of use (p = 0.0937) and ease of learning (p = 0.0036) between Facebook and EasiSocial after the first week of use.

Brandenburgh et al. (2014)	Pre post study	7	65+	Home	Loneliness (DJGLS)	all participants but one had lower loneliness scores than before.
Buhr et al. (2017)	Mixed methods	7 (6 older adults, 1 younger of age 34)	60+ Mean age of older adults 65.1	Home	Usage	-Participants expressed an interest to use this platform to interact with other individuals with aphasia.<br>-Participants also willing to have universally accepted social networking platform to interact with family and friends.
Caroux et al. (2017)	Quasi experiment	26 (13 – Experiment, 13 – control)	65+	Home	Usability over the period of 6 months and 9 months<br><br>Self-perceived health	-usability enhanced across time<br><br>-no significant effect on health
Casey et al. (2020)	Qualitative Interview	107 - people with dementia (n = 38), relatives/carers (n = 28), formal carers (n = 28) and managers (n = 13)	55+	Hospital (n=60), Long-term care (n=29) and Community (n=18)	Perceptions and experience regarding the use and impact of robot MARIO	- most participants across all sites had positive perceptions of, and attitudes towards Robot MARIO.<br>- Across all three settings, all participants suggested that the main impact of MARIO for people with dementia was increased cognitive engagement, autonomy, reduced loneliness, and isolation, all of which led to some improvement in their quality of life
Coelho et al. (2017)	Mixed methods (Log data collected from application, Questionnaire and interview)	3	65+ (Mean age-68.3)	Home	Social interactions,<br>Usability (SUS),<br>User experience (UEQ)	- number of social interactions increases with time,<br>- usability and user experience rated high by participants
Correia et al. (2016)	Controlled Lab study – younger adults<br>5 sueca games	60	Mean age – 24.31	Lab	Trust	-participants who already interacted with the robot increased their level of trust in the following games. Development of trust needs longer interactions
	Questionnaire - younger adults	17	Mean age – 22.62	Sueca Tournament	Usability	-majority answered that they “loved the experience” (64,7%).

Czaja et al. (2018)	RCT	224 (105 – Control, 119 – PRISM)	65+ Intervention mean age – 76.9, control mean age – 75.3	Home	Social isolation (Friendship scale),<br>Loneliness (UCLA scale),<br>social support,<br>social network size (lubben social network index)<br>changes in health-related quality of life and wellbeing,<br>Attitude toward technology, perceptions of the usefulness and usability	Significant decline in social isolation and loneliness, increase in social support and wellbeing.<br>Increase in computer efficacy and computer proficiency.<br>Most participants found PRISM useful and easy to use.
Doppler et al (2018)	Mixed methods (Log data and questionnaire)	30	50+	Home	Usability	Easy to use (86.7% strongly agree and 13.3% agree)
Fields et al. (2019)	Pre post study	15	65+ (mean age - 85.80)	Facility centre	Mood (Face scale),<br>Loneliness (UCLA scale)<br>Depression (GDS-15)	participants reported improvements in mood, loneliness, and depression. The degree of difference/change was slightly greater in participants without dementia
Gao et al. (2015)	Qualitative interview	100	55+ (mean age – 67.1)	University Lab and Residents committee meeting room	Acceptance of application	Two thirds of the participants indicated a willingness to adopt the technology. 42 participants expressed a strong will to use the system.
Garattini et al. (2012)	Mixed methods (Questionnaire and focus group)	19	65+	Home	Social connectedness and system usage (system questionnaire)	-increased social connections and created interactions<br>-easy to use (76%)
Gomes et al. (2014)	Comparative study, Mixed methods (Tasks and Interview)	10	65+ (mean age - 72)	Home	Usability	Prototype provides the older adults with high levels of easiness and satisfaction when compared to Facebook application

Goumopoulos et al. (2017)	Mixed methods (Questionnaires and interview)	20	60+ (mean age - 65.7)	Home	Usability and Acceptability (TAM3) Loneliness (R-UCLA)	Participants rated positively for Usability and acceptability<br>-moderate improvements in loneliness (37.40 (±7.21) to 36.37 (±7.85) with p = 0.034
Isaacson et al. (2019)	Pre post study	40	75+ (mean age – 85.86)	Home	Loneliness (UCLA),<br>Social engagement (Lubben social network scale),<br>Depression (PHQ9),<br>Emotional wellbeing (MHC-SF).	Participants exhibited less loneliness and increased social engagement. Participant's depression was reduced, and also their emotional wellbeing was improved.
Jansen-Kosterink et al. (2020)	Quasi experiment	41	60+ Mean age – 73.4	Home	Loneliness (DGJLS),<br>Quality of life (SF-12v1)<br>Usability (SUS),<br>End user experience (TAM)	No significant change in loneliness (only 31% of users showed a decrease in loneliness),<br>Change in quality of life was positive<br>Usability was acceptable (SUS score 65.3) and 59% of participants willing to continue using the system
Kleinberger et al. (2019)	Mixed methods (Focus group and questionnaire)	10 older adults and 31younger adults (18-54 years old)	Older adults 70+	Senior centre	Technology adoption	Elders saw immense potential for two distinct kinds of connectedness leveraged through the Memory Music Box: 1) Interfamilial Connectedness and 2) Interpersonal Connectedness<br>-positive feedback about the memory box<br>- younger participants felt the device was user friendly for both themselves and their grandparents.
Koceski & Koceska (2016)	Mixed methods (Tasks and Questionnaire)	30 older adults and 5 professional caregivers	65+	Nursing home	User perceptions and acceptance (TAM)	The video conference application was perceived as more useful by elderly group (M = 4.06, SD = 0.98), compared to the caregiver's group (M = 3, SD = 0.71). From elderlies' point of view, this application will reduce the loneliness, by bridging distances, and facilitating communications with friends and family.

Lee et al. (2015)	Mixed Methods (Data log from application, feedback)	15	55+ (mean age- 66.27)	Home	Usage	Participants showed interest to use the fridgenet application and built a virtual community. Also, participants liked the idea of Buy2+gether service.
Machesney, Wexler, Chen & Coppola, (2014)	Mixed Methods (Questionnaire and observation)	13	65+	Home	Loneliness	Loneliness was reduced - evidenced by the observation of participant's positive change in attitude and demeanour.
Marcelino et al. (2016)	Mixed Methods (Tasks and Interview)	23 (20 – older adults and 3 – younger adults)	50+ (mean age – 73.25) 3 younger adults aged 30+ (mean age 35)	Home	Usability	Participants expressed positive feedback and willing to use the system
Morganti et al. (2016)	Formative – Mixed Methods	10 Older adults and 53 Children	OA(M-66.5), C(M-11.6)	School Lab	Usability (CSUQ)	Both older adults and children gave high score for usability. Application generally perceived as usable and was well-accepted among older participants
	Summative - RCT	34 Older adults (OA) and 123 children (C) Control – 19 OA,73 C distributed to 10 teams Intervention – 15 OA,53 C distributed to 10 teams.<br><br>Only OA from both groups participated in psychosocial questionnaire and focus group	OA(M-72), C(M-11.3)	School Lab	Loneliness (ILS), Self-esteem, Engagement (Flow state scale)	-Decrease in emotional loneliness and an improvement in social loneliness; however, values for general loneliness did not decrease in either group. Also, no difference was found in the feeling of loneliness between control and experimental condition.<br>-Self-esteem values increased only for the control condition.<br>-Engagement and involvement were high in both groups and no significant difference between control and experiment group

Muñoz et al. (2015)	Mixed Methods (Tasks and Interview)	9	60+ (mean age – 73.8)	Home	Usability	Participants perceived that the application is usable and useful. Also, participants liked the system and willing to use the system in the future
Muuraiskangas et al. (2012)	Mixed Methods (Tasks, Interview and workshop)	13	65+	Care centre	User experience and acceptance	The overall acceptance of the current system was rather low. Only three participants (three from the one-on-one sessions, none from the workshop) could imagine using the system in the future if it were improved, and one was determined not to use such a system in any case
Neves, Franz, Judges, Beermann, & Baecker (2019)	Mixed Methods (Questionnaire, observation and Interview)	12	70+ (mean age 82.5)	Retirement home	Social support (Duke social support index scale), Loneliness (UCLA), Acceptability	Although the app increased sense of social interactions (communication frequency and type) with family and friends for 10 participants, only four reported high perceived social connectedness at post deployment. No significant changes in both social support and loneliness.<br>-acceptability of the app based on various socio technical factors
Pereira et al. (2015)	Mixed Methods (Tasks and Questionnaire)	7	75+ (mean age – 83)	Nursing home	Usability	Participants found it useful and would like to use it in future.
Restyandito et al. (2020)	Mixed Methods (Tasks and Questionnaire)	30	60+ (mean age 73.2)	Did not mention	Usability (UEQ)	UEQ score was found satisfactory where attractiveness, perspicuity, efficiency, dependability,<br>stimulation and novelty received an excellent score.
Romanyk et al. (2015)	Pilot – preliminary test	12	70+ (mean age 77.67)	Did not mention	Usefulness	Participants interested in this idea, and they desired for the single-content interface (which most closely mimics a traditional television channel).
Scandurra & Sjölinder (2013)	Codesign - 10 evaluation workshops - Qualitative	8	65+	Home	User experience and Acceptance	-it was easy to use<br>- participants felt it was something new and useful<br>- participants wanted to continue using it.

Sidner et al. (2018)	Quasi experiment	44(11 control, 24 virtual agent, 9 robot)	55+ (Mean age 66)	Home	Social support (Social support questionnaire), Loneliness (UCLA), Health (SF-12)	-No significant changes in participants health or relationship status across all three conditions.
Tapia et al. (2016)	Heuristic Evaluation – Experts	3	NA	Lab	Usability	Experts suggested usability improvements which are then implemented and evaluated further with older adults
	Usability Tests (thinking aloud) -older adults	8	60+	Lab (Simulated as home)	Usability	Older adults perceived the application as useful but not confident of using the system without external assistance.
Tullius & Dogan (2020)	Mixed methods	5	60+ (mean age – 66.2)	did not mention	Technology readiness (Technology commitment questionnaire) and usability (UEQ)	-most participants showed positive opinion towards using new technology<br>-easy and understandable to use, and would like to use it in future
Zaine et al. (2019)	2 case study - Interview	Family case study -3<br>Friendship case study - 3	60+	Home	User experience (UEQ), Usability (SUS)<br>Social connectedness	-Both case study revealed positive social effects for both deepening and developing of relationships.<br>- Both study participants rated very high scores for user experience and usability.<br>- All participants reported feeling closer to each other and contacting each other more than usual
Zhao et al. (2016)	Qualitative interview	200	did not mention	Exhibition	Usability (usefulness)	both the older adults and young generation are quite interested in, felt useful and willing to have a try on Blossom.
Zuckerman et al. (2020)	Quasi experiment	39 (Companion-Function – 13 Game-Function - 13, No-Function - 13)	Mean age – 75	University lab	Participant's perception and acceptance (Robot opinions questionnaire)	- participants in all conditions associated the non-verbal gestures with a social context and especially associated with the feelings of being seen.<br>-participants gave higher acceptance rating for game function.

RCT-Randomised Control Trial, SUS -System Usability Scale, UCLA - University of California, Los Angeles, DJGLS - De Jong Gierveld Loneliness Scale, UEQ – user experience questionnaire, GDS – Geriatric depression scale, TAM3 – Technology Acceptance Model 3, R-UCLA – Revised University of California, Los Angeles, PHQ9- Patient Health Questionnaire 9, MHC-SF -Mental Health Continuum- Short Form,SF-12v1 – 12 item Short form questionnaire version 1, CSUQ – Computer System Usability Questionnaire, ILS – Italian Loneliness Scale.
